# Identification of Diverse Bat Alphacoronaviruses and Betacoronaviruses in China Provides New Insights Into the Evolution and Origin of Coronavirus-Related Diseases

**DOI:** 10.3389/fmicb.2019.01900

**Published:** 2019-08-14

**Authors:** Yelin Han, Jiang Du, Haoxiang Su, Junpeng Zhang, Guangjian Zhu, Shuyi Zhang, Zhiqiang Wu, Qi Jin

**Affiliations:** ^1^NHC Key Laboratory of Systems Biology of Pathogens, Institute of Pathogen Biology, Chinese Academy of Medical Sciences and Peking Union Medical College, Beijing, China; ^2^Key Laboratory of Zoonosis of Liaoning Province, College of Animal Science and Veterinary Medicine, Shenyang Agricultural University, Shenyang, China; ^3^EcoHealth Alliance, New York, NY, United States

**Keywords:** bats, coronaviruses, porcine epidemic diarrhea virus, severe acute respiratory syndrome coronavirus, ecological and genetic diversity

## Abstract

Outbreaks of severe acute respiratory syndrome (SARS) in 2002, Middle East respiratory syndrome in 2012 and fatal swine acute diarrhea syndrome in 2017 caused serious infectious diseases in humans and in livestock, resulting in serious public health threats and huge economic losses. All such coronaviruses (CoVs) were confirmed to originate from bats. To continuously monitor the epidemic-related CoVs in bats, virome analysis was used to classify CoVs from 831 bats of 15 species in Yunnan, Guangxi, and Sichuan Provinces between August 2016 and May 2017. We identified 11 CoV strains from 22 individual samples of four bat species. Identification of four alpha-CoVs from *Scotophilus kuhlii* in Guangxi, which was closely related to a previously reported bat CoV and porcine epidemic diarrhea virus (PEDV), revealed a bat-swine lineage under the genus *Alphacoronavirus*. A recombinant CoV showed that the PEDV probably originated from the CoV of *S. kuhlii*. Another alpha-CoV, α-YN2018, from *Rhinolophus sinicus* in Yunnan, suggested that this alpha-CoV lineage had multiple host origins, and α-YN2018 had recombined with CoVs of other bat species over time. We identified five SARS-related CoVs (SARSr-CoVs) in *Rhinolophus* bats from Sichuan and Yunnan and confirmed that angiotensin-converting enzyme 2 usable SARSr-CoVs were continuously circulating in *Rhinolophus* spp. in Yunnan. The other beta-CoV, strain β-GX2018, found in *Cynopterus sphinx* of Guangxi, represented an independently evolved lineage different from known CoVs of *Rousettus* and *Eonycteris* bats. The identification of diverse CoVs here provides new genetic data for understanding the distribution and source of pathogenic CoVs in China.

## Introduction

Coronaviruses (CoVs) are a group of enveloped viruses with a large positive single-stranded RNA genome (∼26–32 kb in length) of the subfamily *Coronavirinae* under the family *Coronaviridae*. The complete genome of CoV contains five major open reading frames (ORFs) that encode replicase polyproteins (ORF1ab), spike glycoprotein (S), envelope protein (E), membrane protein (M), and nucleocapsid protein (N) flanked by a 5′- untranslated region (UTR) and a 3′- UTR. Currently, members of the subfamily Coronavirinae are classified into four genera, *Alphacoronavirus*, *Betacoronavirus*, *Gammacoronavirus*, and *Deltacoronavirus* (Fehr and Perlman, 2015; Su et al., 2016). CoVs can cause upper and lower respiratory diseases, gastroenteritis, and central nervous system infections in a wide variety of avian and mammalian hosts. Some CoVs are human pathogens that cause mild to severe disease, including NL63 and 229E of the genus *Alphacoronavirus*, and severe acute respiratory syndrome CoV (SARS-CoV), Middle East respiratory syndrome CoV (MERS-CoV), OC43, and HKU1 of the genus *Betacoronavirus* (Dijkman and van der Hoek, 2009; Desforges et al., 2014; van den Brand et al., 2015; Lim et al., 2016). The SARS pandemic originated in Guangdong Province in China between 2002 and 2003, and spread to 29 countries, resulting in nearly 8,000 cases and 800 deaths worldwide (Donnelly et al., 2004). Since being discovered in Middle Eastern countries in 2012, MERS-CoV has infected 2,260 people with a current fatality rate of 35.5% (Mackay and Arden, 2015; van den Brand et al., 2015; de Wit et al., 2016).

With the discovery of diverse wildlife-borne CoVs indifferent regions of the world, previous studies have indicated that bats are the main and original natural reservoirs of *Alphacoronavirus* and *Betacoronavirus* (Calisher et al., 2006; Woo et al., 2012). The special metabolic and immune systems allow bats to tolerate diverse viruses, and the social roosting behavior of many bat species lets them serve as ideal incubators for the occurrence of frequent co-infection, recombination, and intra-species transmission of CoVs (Zhang et al., 2013; O’Shea et al., 2014; Hu et al., 2015; Lu et al., 2015; Sola et al., 2015). Although 15 years have passed without a recurrence of the SARS outbreak, the consistent discovery of SARS-related CoVs (SARSr-CoVs) in *Rhinolophus* bats indicates an ongoing risk of SARS re-emergence (Li et al., 2005; Balboni et al., 2012; Yang et al., 2013; Wu et al., 2016b; Hu et al., 2017). Recently, a CoV of *Rhinolophus* bat origin, swine acute diarrhea syndrome CoV (SADS-CoV), was found to be responsible for the death of 24,693 piglets across four farms in Guangdong Province (Zhou et al., 2018). Besides similar clinical signs, the outbreak of SADS coincided with infection by another swine enteric CoV, porcine epidemic diarrhea virus (PEDV; Zhou et al., 2018). It is worth mentioning that a PEDV-related bat CoV, BtCoV/512, was previously identified in *Scotophilus kuhlii* bats (Tang et al., 2006; Wang et al., 2016; Gerdts and Zakhartchouk, 2017; Zhou et al., 2018). All of these findings emphasize the importance of continuous monitoring for ecological diversity of CoVs in bats to minimize the impact of potential CoV-related diseases on public health and economic growth.

As the original site of the SARS epidemic in humans and SADS outbreak in pigs (Heymann et al., 2013), Guangdong Province is also the primary region in China in which wildlife is being consumed. Since the supply of wildlife for consumption in Guangdong mainly comes from Yunnan and Guangxi Provinces (Wu et al., 2016b), understanding the presence and dynamic changes in SARS- or SADS- related bat CoVs within and between these provinces is important for predicting and tracing CoV-related diseases. Sichuan is next to Yunnan Province, and has high ecological diversity of animal resources, and it is the largest pig-farming province in China. Although PEDV-related pig diseases were reported previously (Cai et al., 2016), no systematic survey of bat-borne CoVs has been conducted in Sichuan to establish if there are SARS- or SADS- related CoVs.

In this study, a survey of bat-borne CoVs was performed in bats of Guangxi, Yunnan, and Sichuan provinces between 2016 and 2017. Samples from *Rhinolophus* bats in Sichuan and Yunnan Provinces, and *S. kuhlii* and *Cynopterus sphinx* in Guangxi Province were found to be CoV-positive, and a large number of sequencing reads classified into diverse alpha- and beta-CoVs were obtained using virome analysis. The characterization of 11 CoVs in bats from different regions provides clues for the evolutionary relationships of PEDV, SARS-CoV, and other bat-associated CoVs.

## Materials and Methods

### Bat Sampling

Bats were treated according to the guidelines of Regulations for the Administration of Laboratory Animals (Decree No. 2 of the State Science and Technology Commission of the People’s Republic of China, 1988). The sampling was approved by the Ethics Committee of Institute of Pathogen Biology, Chinese Academy of Medical Sciences & Peking Union Medical College (Approval number: IPB EC20100415). The bat species were initially determined morphologically and subsequently confirmed by sequence analysis of mitochondrial cytochrome b DNA (Tang et al., 2006; Du et al., 2016). Anal swab samples from captured bats were immersed in virus sampling tubes (Yocon, China) containing maintenance medium and temporarily stored at −20°C. The samples were then transported to the laboratory and stored at −80°C. The accurate sampling locations were recorded by place name, latitude, and longitude.

### Viral DNA and RNA Library Construction and Next-Generation Sequencing

The samples of each species were pooled by adding 1 ml from each maintenance medium sample into one new sample tube. The pooled samples, classified by species, were processed with a virus-particle-protected, nucleic acid purification method as described in our previous studies (Wu et al., 2012, 2016a, 2018). The samples were homogenized and subsequently filtered through a 0.45 μm polyvinylidene difluoride filter (Millipore, Germany). The filtered samples were then centrifuged at 150,000 × *g* for 3 h at 4°C. To remove naked DNA and RNA, the pellet was digested in a cocktail of DNase and RNase enzymes. The viral DNA and RNA were simultaneously isolated using a QIAmp MinElute Virus Spin Kit (Qiagen, United States). First-strand viral cDNA was synthesized using the primer K-8N and a Superscript III system (Invitrogen, United States). The cDNA was converted into dsDNA by Klenow fragment (NEB, United States). Sequence-independent PCR amplification was conducted using primer K. The PCR products were analyzed by agarose gel electrophoresis. All DNA smears larger than 500 bp were extracted by a MinElute Gel Extraction Kit (Qiagen). The extracted nucleic acid libraries were then analyzed using an Illumina HiSeq2500 sequencer, for a single read of 100 bp. The sequence reads were filtered using previously described criteria (Wu et al., 2012, 2016a, 2018). Reads with no call sites, reads with similarity to the sequencing adaptor and the primer K sequence, duplicate reads, and low-complexity reads were removed.

### Taxonomic Assignment

Sequence-similarity-based taxonomic assignments were conducted as described in our previous study (Wu et al., 2012). Valid sequence reads were aligned to sequences in the NCBI non-redundant nucleotide database and non-redundant protein database using BLASTn and BLASTx, respectively. The taxonomies of the aligned reads with the best BLAST scores (E score <10^–5^) were parsed by the MEGAN 6 - MetaGenome Analyzer.

### Viral Prevalence and Genome Sequencing

CoV screening of individual samples was performed by amplifying a 440-bp fragment of the RNA-dependent RNA polymerase (RdRp) gene of CoVs using conserved primers (5′-GGTTGGGACTATCCTAAGTGTGA-3′ and 5′-CCATC ATCAGATAGAATCATCATA-3′), as described previously (Du et al., 2016).

Sequence reads classified into the same virus genus were extracted. The accurate locations of the reads were determined based on the alignment results exported with MEGAN 6. Specific primers were designed from the located sequence reads. Fragments between reads were amplified with nested specific primers and then sequenced. The remaining genomic sequences were determined using 5′- and 3′- rapid amplification of cDNA ends.

### Phylogenetic and Recombination Analysis

MEGA6.0 was used to align nucleotide sequences and deduced amino acid sequences using the MUSCLE package and default parameters. The best substitution model was then evaluated by the Model Selection package. Finally, we constructed a maximum-likelihood method using an appropriate model to process the phylogenetic analyses with 1,000 bootstrap replicates. Recombination among CoVs was detected with SimPlot software. All analyses were performed with a Kimura model, a window size of 1000 bp, and a step size of 100 bp.

### Nucleotide Sequence Accession Numbers

All genome sequences were submitted to GenBank. The accession numbers for the five bat alpha-CoVs were MK211369 to MK211373. The accession numbers for the six bat beta-CoVs were MK211374 to MK211379.

## Results

### Virome and Prevalence Analysis of CoVs

**FIGURE 1:**
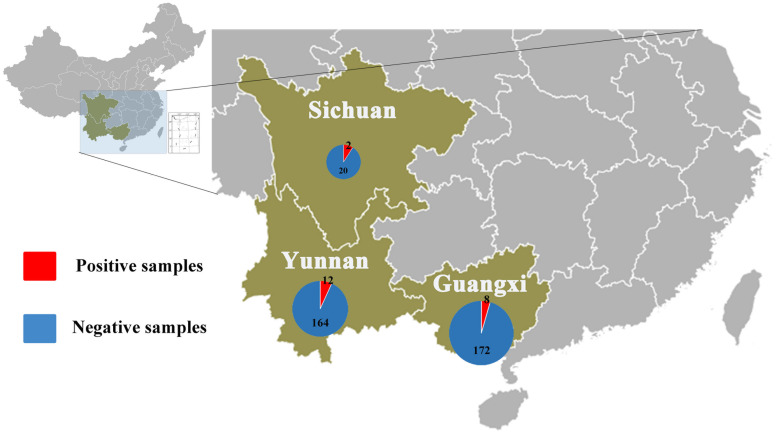
Numbers of bat samples from Sichuan, Yunnan, and Guangxi Provinces. The size of the pie chart is in proportion with the total number of specimens collected from each province. Red color represents samples positive for CoV, and blue color represents negative samples.

Anal swabs from 831 bats of 15 species were collected from Yunnan, Guangxi, and Sichuan Provinces in China between August 2016 and May 2017. All anal swab samples were classified by species and then combined into 27 pools and subjected to virome analysis. CoV-related sequencing reads were detected in ten of the 27 sample pools. These 10 pools related to 22 *Rhinolophus* spp. samples from Sichuan, 176 *Rhinolophus sinicus* samples from Yunnan, 150 *S. kuhlii* samples from Guangxi, and 30 *C. sphinx* samples from Guangxi. Pan CoV screening revealed that 22 individual samples were CoV-positive (Table 1 and Figure 1). A total of 1,040,901 reads of 100 bp in length showed the best matches with *Coronavirinae* viral proteins in the NCBI non-redundant database, and these reads showed 40–100% amino acid (aa) identity with known alpha- or beta-CoVs.

**TABLE 1:** Coronavirus distribution in different bat species and locations.

Province	Species	Pool code for NGS	Collection date [year.month]	Virus detection rate (pos./indiv. [%])	Sequencing reads related to CoV	Virus
Sichuan	*Rhinolophus* spp.	55	2016.08	2/22 [9.1%]	1598	BtRl-BetaCoV/SC2018
Yunnan	*Rhinolophus sinicus*	57	2016.09	2/39 [5.1%]	84818	BtRs-BetaCoV/YN2018A
	*Rhinolophus sinicus*	60	2016.09	4/40 [10%]	884839	BtRs-BetaCoV/YN2018B BtRs-AlphaCoV/YN2018
	*Rhinolophus sinicus*	61	2016.09	2/46 [4.3%]	37997	BtRs-BetaCoV/YN2018C
	*Rhinolophus sinicus*	62	2016.09	4/51 [7.8%]	11131	BtRs-BetaCoV/YN2018D
Guangxi	*Scotophilus kuhlii*	3	2017.05	2/27 [7.4%]	5609	BtSk-AlphaCoV/GX2018A
	*Scotophilus kuhlii*	4	2017.05	1/39 [2.6%]	4381	BtSk-AlphaCoV/GX2018D
	*Cynopterus sphinx*	11	2017.05	2/30 [6.7%]	6138	BtCs-BetaCoV/GX2018
	*Scotophilus kuhlii*	12	2017.05	1/41 [2.4%]	4353	BtSk-AlphaCoV/GX2018B
	*Scotophilus kuhlii*	13	2017.05	2/43 [4.7%]	37	BtSk-AlphaCoV/GX2018C

Eleven viruses of representative positive samples were selected for genomic sequencing as quasi-species. There were five viruses in the genus *Alphacoronavirus*; four from *S. kuhlii* in Guangxi Province were named as BtSk-AlphaCoV/GX2018A (α-GX2018A), BtSk-AlphaCoV/GX2018B (α-GX2018B), BtSk-AlphaCoV/GX2018C (α-GX2018C), and BtSk-AlphaCoV/GX2018D (α-GX2018D). The other virus from *R. sinicus* in Yunnan Province was named as BtRs-AlphaCoV/YN2018 (α-YN2018). There were six CoVs in the genus *Betacoronavirus*. The virus identified from *Rhinolophus* spp. in Sichuan Province was named as BtRl-BetaCoV/SC2018 (β-SC2018); four CoVs from *R. sinicus* in Yunnan Province were named as BtRs-BetaCoV/YN2018A (β-YN2018A), BtRs-BetaCoV/YN2018B (β-YN2018B), BtRs-BetaCoV/YN2018C (β-YN2018C), and BtRs-BetaCoV/YN2018D (β-YN2018D). One CoV from *C. sphinx* in Guangxi Province was named as BtCs-BetaCoV/GX2018 (β-GX2018).

### Genome Organization

**FIGURE 2:**
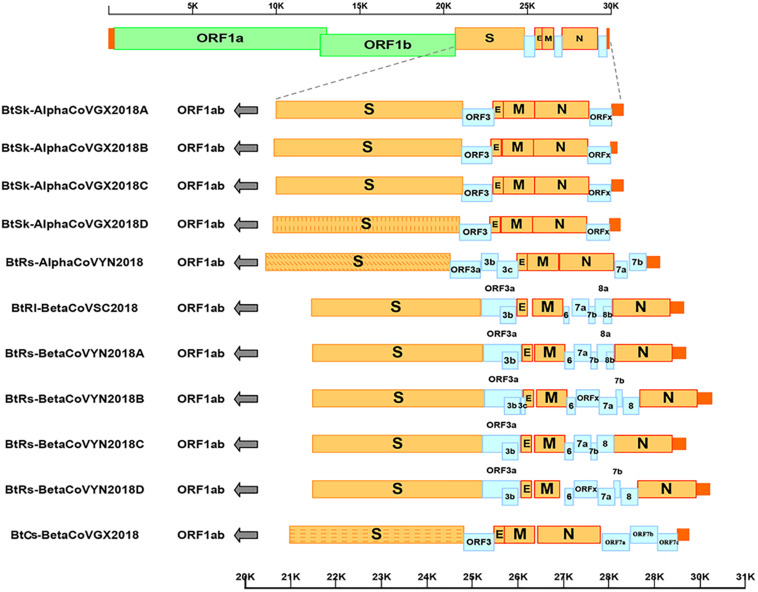
Genome analysis of eleven novel CoVs. Color-coding for different genomic regions as follows. Green, non-structural polyproteins ORF1a and ORF1b; yellow, structural proteins S, E, M and N; blue, accessory proteins ORF3, ORF6, ORFx, ORF7 and ORF8; orange, untranslated regions.

As shown in Table 2 and Figure 2, with the typical genome organization, the full genome sizes of 11 CoVs ranged from 28, 146 (α-GX2018C) to 30, 256 bases (β-YN2018B) and their G + C contents ranged from 0.38 to 0.41. There were several accessory ORFs in each CoV strain. These accessory ORFs were mainly present in the regions between S and E (named ORFs-e), M and N (named ORFm-n), or downstream of N (named ORFn). The accessory ORFs of α-GX2018 (A–D), α-YN2018, and β-GX2018 were mainly present in ORFs-e and ORFm-n regions; the small ORFs of β-SC2018 and β-YN2018 (A–D) were mainly present in ORFs-e and ORFm-n regions. We named these accessory ORFs according to previous studies.

**TABLE 2:** Predicted ORFs in the genomes of bat CoVs.

**Coronavirus**			**Alphacoronavirus**					**Betacoronavirus**					
			***Scotophilus kuhlii***				***Rhinolophus* spp.**						***Cynopterus sphinx***
			**α-GX2018A**	**α-GX2018B**	**α-GX2018C**	**α-GX2018D**	**α-YN2018**	**β-SC2018**	**β-YN2018A**	**β-YN2018B**	**β-YN2018C**	**β-YN2018D**	**β-GX2018**
Genome size (nt)			28,303	28,175	28,146	28,235	29,109	29,648	29,698	30,256	29,689	30,213	29,752
G + C content			0.4	0.4	0.4	0.4	0.41	0.41	0.41	0.41	0.41	0.41	0.38
5’ UTR	Location	(nt)	1–295	1–259	1–255	1–247	1–324	1–260	1–265	1–263	1–262	1–264	1–225
ORF1ab	Location	(nt)	296–20,676	260–20,640	256–20,636	248–20,631	328–20,468	261–21,448	266–21,219	264–21,481	263–21,483	265–21,485	226–21,032
	Length	(nt)	20,381	20,381	20,381	20,384	20,144	21,188	20,954	21,218	21,221	21,221	20,807
		(aa)	6,793	6,793	6,793	6,794	6,714	7062	6985	7,072	7,073	7,073	6,935
S	Location	(nt)	20,673–24,788	20,637–24,755	20,633–24,751	20,622–24,719	20,465–24,517	21,455–25,180	21,494–25,222	21,491–25,258	21,490–25,215	21,492–25,217	20,980–24,813
	Length	(nt)	4,119	4,119	4,119	4,098	4,053	3726	3729	3,768	3,726	3,726	3,834
		(aa)	1,371	1,371	1,371	1,366	1,350	1,241	1,240	1,255	1,241	1,241	1,277
ORFs-e	Location	(nt)	24,788–25,462	24,755–25,429	24,751–25,425	24,719–25,399	24,517–25,565	25,190–25,955	25,232–26,056	25,270–26,094	25,225–26,049	25,227–26,051	24,810–25,472
	Number	(nt)	1	1	1	1	3	2	2	2	2	2	1
E	Location	(nt)	25,443–25,673	25,410–25,640	25,406–25,636	25,380–25,616	25,977–26,201	26,039–26,269	26,081–26,311	26,119–26,349	26,074–26,304	26,076–26,306	25,472–25,717
	Length	(nt)	231	231	231	237	225	231	231	231	231	231	246
		(aa)	76	76	76	79	74	76	76	76	76	76	81
M	Location	(nt)	25,680–26,366	25,647–26,333	25,643–26,329	25,623–26,315	26,213–26,896	26,320–26,985	26,362–27,027	26,400–27,065	26,355–27,020	26,357–26,914	25,704–26,372
	Length	(nt)	678	678	678	693	684	666	666	666	666	558	669
		(aa)	225	225	225	231	227	221	221	221	221	185	222
ORFm-n	Location	(nt)	—	—	—	—	—	26,835–28,061	27,038–28,108	27,076–28,668	27,031–28,101	27,032–28,624	—
	Number	(nt)	—	—	—	—	—	5	5	5	4	5	—
N	Location	(nt)	26,377–27,561	26,344–27,528	26,340–27,524	26,326–27,504	26,907–28,100	28,076–29,341	28,123–29,391	28,683–29,951	28,116–29,384	28,639–29,907	26,427–27,818
	Length	(nt)	1,185	1,185	1,185	1179	1,194	1,266	1,269	1,269	1,269	1,269	1,392
		(aa)	394	394	394	393	397	421	422	422	422	422	463
ORFn	Location	(nt)	27,576–28,070	27,543–28,037	27,539–28,033	27,519–28,010	28,141–28,817	—	—	—	—	—	27,854–29,510
	Number	(nt)	1	1	1	1	2	—	—	—	—	—	3
3’ UTR	Location	(nt)	28,071–28,291	28,038–28,175	28,034–28,146	28,011–28.235	28,817–29,098	29,342–29,648	29,392–29,698	29,952–30,256	29,385–29,689	29908–30213	29,511–29,741
													

As shown in Table 3, The core sequences of the leader transcriptional regulatory sequence (TRS; 5′-CUAAAC-3′) were identified in the 5′ untranslated sequences of α-GX2018 (A–D) and α-YN2018, which is unique to *Alphacoronavirus* (Madhugiri et al., 2014). The TRS motifs of S, ORF3, E, and M genes in α-GX2018 (A-D) differed from the core sequences of the leader TRS (Supplementary Table 1). The TRS motif of S was identified as 5′-CCAAAU-3′ or 5′-CCAAAC-3′; the TRS motif of ORF3 was identified as 5′-CAUUAC-3′; the TRS motif of E was identified as 5′-CUAGAC-3′; the TRS motif of M was identified as 5′-AUAAAC-3′. An alternative TRS motif (5′-GUAAAC-3′) of α-YN2018 was discovered preceding ORF3, which differed by 1 nucleotide (nt) from those of all other genes in α-YN2018. In addition to the E of β-GX2018, the TRS motifs of six betacoronaviruses all conformed to the consensus motif 5′-ACGAAC-3′, which is unique to *Betacoronavirus* (Woo et al., 2010). An alternative TRS motif (5′-UCGAAC-3′) was discovered preceding the E in β-GX2018.

**TABLE 3:** Transcription regulatory sequences (TRS) for four representative bat CoVs^a,b^.

				**Leader TRS region and**	**Distance**
**Coronavirus**	**ORF**		**TRS location (CoV) (nt)**	**intergenic TRS (CoV)**	**from TRS to AUG (nt)**
α-GX2018D	ORF1ab		23–28	UUCAA **CUAAAC** GAAAU	219
	S		20,615–20,620	UUCAA **CCAAAC** **AAUG**	1
	ORF3		20,664–20,669	UUCAA **CAUUAC** GAACC	29
	E		25,428–25,433	UUCAA **CUAGAC** GAAU**AUG**	4
	M		25,639–25,644	CAAGU **AUAAAC** GAAA**AUG**	4
	N		26,207–26,212	UUAGU **CUAAAC** AGAAA	8
	ORFx		27,563–27,568	UUCAA **CUAAAA** CA**AUG**	2
α-YN2018	ORF1ab		69–74	CUCAA **CUAAAC** GAAAU	250
	S		20,455–20,460	GUCAA **CUAAAC** UAAA**AUG**	4
	ORF3	a	24,471–24,476	UCGUG **GUAAAC** GUUUA	40
		b			—
		c			—
	E		25,905–25,910	CUCAA **CUAAAC** GACUU	66
	M		26,206–26,211	AUCAA **CUAAAC** A**AUG**	1
	N		26,898–26,903	AUAAA **CUAAAC** AAC**AUG**	3
	ORF7	a	28,123–28,128	AUCAA **CUAAAC** **AUG**	0
		b			—
β-YN2018B	ORF1ab		66–67	UCUAA **ACGAAC** UUUAA	192
	S		21,485–21,490	ACUAA **ACGAAC** **AUG**	0
	ORF3	a	25,262–25,267	CAUAA **ACGAAC** UU**AUG**	2
		b			—
		c			—
	E		26,111–26,116	UGAGU **ACGAAC** UU**AUG**	2
	M		26,350–26,355	UCUAA **ACGAAC** UAACUA	44
	ORF6		26,915–26,920	ACAUC **ACGAAC** GCUUU	155
	ORFx		27,271–27,276	GAUAA **ACGAAC** CACU**AUG**	4
	ORF7	a	27,791–27,796	UCUAA **ACGAAC** **AUG**	0
		b			—
	ORF8 a b		28,297–28,302	UCUAA **ACGAAC** **AUG**	0
	N		28,669–28,674	UCUAA **ACGAAC** AAACU	8
β-GX2018	ORF1ab		70–75	CUUGA **ACGAAC** UAAAA	150
	S		20,932–20,937	GUUGA **ACGAAC** UGAUU	42
	ORF3		24,801–24,806	AAUAA **ACGAAC** AGA**AUG**	3
	E		25,462–25,467	CGCCG **UCGAAC** UAUAAUG	4
	M		25,677–25,682	CUUGA **ACGAAC** AAGAA	21
	N		26,416–26,421	UUUGA **ACGAAC** CAAUU**AUG**	5
	ORF7	a	27,847–27,852	CCUUA **ACGAAC** C**AUG**	1
		b	28,474–28,479	CUUGA **ACGAAC** **AUG**	0
		c	29,057–29,062	AGGUU **ACGAAC** AUCUA	7

*a, These four representative bat CoVs are α-GX2018D, α-YN2018, β-YN2018B and β-GX2018, respectively. b, For putative ORFs, we aligned the TRS that preceded the start codon AUG with the leader TRS. The core sequence is indicated with underscores in bold type. The start codons of genes are in bold blue.*

### Sequence Similarity Analysis

#### Four Alpha-CoVs of *S. kuhlii* in Guangxi Province

**FIGURE 3:**
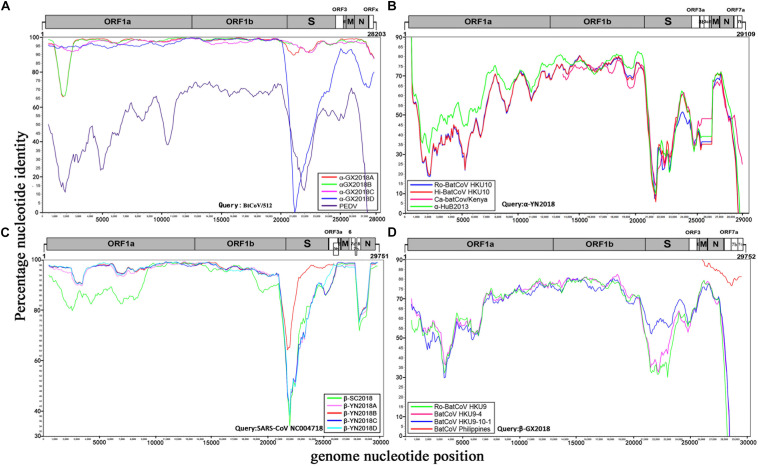
Similarity plot analysis. **(A)** Full-length genome sequence of BtCoV/512 was used as a query sequence and α-GX2018A, B, C, D, and PEDV as reference sequences. **(B)** Full-length genome sequence of α-YN2018 was used as a query sequence and Ro-BatCoV HKU10, Hi-batCov HKU10, Ca-batCov/Kenya, and α-HuB2013 as reference sequences. **(C)** Full-length genome sequence of SARS-CoV SZ3 was used as a query sequence and β-YN2018A, B, C, D and β-SC2018 as reference sequences. **(D)** Full length genome sequence of β-GX2018 was used as query sequence and BatCoV HKU9-4, BatCoV HKU9-10-1, Ro-BatCoV HKU9, and BatCoV Philippines/Diliman as reference sequences. These analyses were performed with the Kimura model, a window size of 1000 base pairs and a step size of 100 base pairs.

Four alpha-CoVs were identified from *S. kuhlii*. The full-length genomes of strains α-GX2018A, B, C, and D were sequenced. The genome sequence similarity among these four CoVs, PEDV, and BtCoV/512 was examined by Simplot analysis (Figure 3A) and pairwise alignment (Supplementary Table 2). The α-GX2018A, B, and C were closely related to a known CoV, BtCoV/512, identified in *S. kuhlii* of Hainan Province with 96.9–100% aa identity for ORF1b, ORF3, E, M, and N. The differences mainly occurred in S (93.4–96.1% aa identity) and ORFx (65.7–67.6% aa identity). There were also significant differences between α-GX2018A and B and BtCoV/512 at the front end of ORF1a (1000–2500 nt) with 81.3–82% nt identity. In addition, although the α-GX2018D was closely related to that of BtCoV/512 with 92.2–99.4% aa identity for ORF1a, ORF1b, ORF3, and M, it was unexpected that the aa identity of α-GX2018D and BtCoV/512 in S1 region was 39.7%, which was lower than that of α-GX2018D and PEDV (42.7% aa identity). Although the aa identity of α-GX2018D and PEDV in S1 region was only 42.7%, we have not found any known viruses with higher identity than this.

#### Novel Alpha-CoV of *R. sinicus* in Yunnan Province

A novel alpha-CoV (α-YN2018) was identified in*R. sinicus* of Yunnan Province. The genome sequencesimilarity among α-YN2018, BtRf-alphaCoV/HuB2013 (α-HuB2013), *Hipposideros* bat CoV HKU10 (Hi-batCov HKU10), *Rousettus* bat CoV HKU10 (Ro-BatCoV HKU10), and *Cardioderma* bat coronavirus/Kenya/KY43/2006 (Ca-batCov/Kenya) was examined by Simplot analysis (Figure 3B) and pairwise alignment (Supplementary Table 3). This virus showed low sequence similarity with any known alpha-CoVs. α-YN2018 showed the highest sequence similarity to α-HuB2013 of *Rhinolophus ferrumequinum* with 63.9–92.4% aa identity in ORF1a, ORF1b, and N; to Hi-batCov HKU10 with 50–85.7% aa identity in S, M, and ORF7b; to Ca-batCov/Kenya in ORF3a with 60.6% aa identity; and to Ro-BatCoV HKU10 in E with 74.3% aa identity. The sequences of ORF7a from α-YN2018 showed <25% aa identity with those of the afore-mentioned known CoVs. In addition, we did not find any sequences that were similar to ORF3b and ORF3c of α-YN2018 from known viruses. According to the ICTV criteria, CoVs that share <90% aa sequence identity in the conserved replicase domains are considered to belong to different species; thus, α-YN2018 could be considered a new species under the genus ***Alphacoronavirus***.

#### Five Beta-CoVs of *Rhinolophus* spp. From Sichuan and Yunnan Provinces

We obtained five beta-CoVs from *Rhinolophus* spp. from Yunnan and Sichuan Provinces, which were named as β-YN2018A, β-YN2018B, β-YN2018C, β-YN2018D, and β-SC2018. All of these beta-CoVs had high sequence identity with SARSr-CoVs. These five SARSr-CoVs showed 92–97% nt identity with human SARS-CoV SZ3. The genome sequence similarity among the five SARSr-CoVs and SARS-CoV SZ3 strain was examined by Simplot analysis (Figure 3C). These five SARSr-CoVs were highly conserved and shared a uniformly high sequence similarity to SARS-CoV with 94.2–100% aa identity in ORF1a, ORF1b, E, M, and N (Supplementary Table 4). There were some differences between the SARSr-CoVs and SARS-CoV SZ3 in the coding regions of ORF3a, ORF3b, ORF7a, and ORF7b. After further analysis, the SARS-CoV SZ3 showed the highest sequence similarity to β-YN2018B with 92–96% aa identity in S, ORF3a, and ORF3b; and to β-YN2018A with 93.2–95.9% aa identity in ORF7a and ORF7b. In contrast, considerable genetic diversity was shown in the S (72.9–92.4% aa identity) and ORF8 (32.6–34.1% aa identity) among the five SARSr-CoVs and SARS-CoV SZ3 strain.

β-YN2018B shared 98.8% aa identity with SARSr-CoV WIV1, which was higher than that of the other four SARSr-CoVs (78.8–79.1% aa identity) (Figure 3C and Supplementary Table 4). Remarkably, WIV1 and WIV16 (or Rs4874) were identified between 2012 and 2013, and they could use the human angiotensin converting enzyme II (ACE2) receptor for entry, like SARS-CoV (Ge et al., 2013; Yang et al., 2015).

ORFx was also found in the genomes of β-YN2018B and β-YN2018D. ORFx of β-YN2018B and β-YN2018D both shared 98.8% aa identity with WIV1. Except for ORF3b (95.6% aa identity), β-YN2018B was highly conserved and shared a uniformly high sequence similarity to SARS-CoV WIV1 in every ORF (98.2–100% aa identity) (Supplementary Table 4).

#### Novel Beta-CoV of *C. sphinx* in Guangxi Province

A novel beta-CoV, β-GX2018, was identified from this bat species. The genome sequence similarity among β-GX2018 and four known CoVs, BatCoV HKU9-4, BatCoV HKU9-10-1, Ro-BatCoV HKU9, and BatCoV Philippines/Diliman was examined by Simplot analysis (Figure 3D) and pairwise alignment (Supplementary Table 5). The full-length genome sequence of this virus had sequence similarity to that of HKU9- related CoVs of *Rousettus* bats and GCCDC1-related CoVs of *Eonycteris* bats (Woo et al., 2007; Huang et al., 2016), but the sequence identity between them was low. The sequence similarity between β-GX2018 and HKU9- and GCCDC1-related CoV s were 51–90.8% aa identity in ORF1a, ORF1b, S, ORF3, E, M, and N. Some partially sequenced genome segments of a recently reported CoV, Philippines/Diliman1525G2/2008 (BatCoV Philippines/Diliman) (Watanabe et al., 2010), identified in *C. brachyotis* from the Philippines, showed the highest aa sequence identity with the β-GX2018 in M (96% aa identity), N (91% aa identity), ORF7a (80% aa identity), ORF7b (84.3% aa identity), and ORF7c (80.8% aa identity) (Watanabe et al., 2010). According to the ICTV criteria, β-GX2018 could be considered a new species under the genus *Betacoronavirus*.

### Phylogenetic Analysis

**FIGURE 4:**
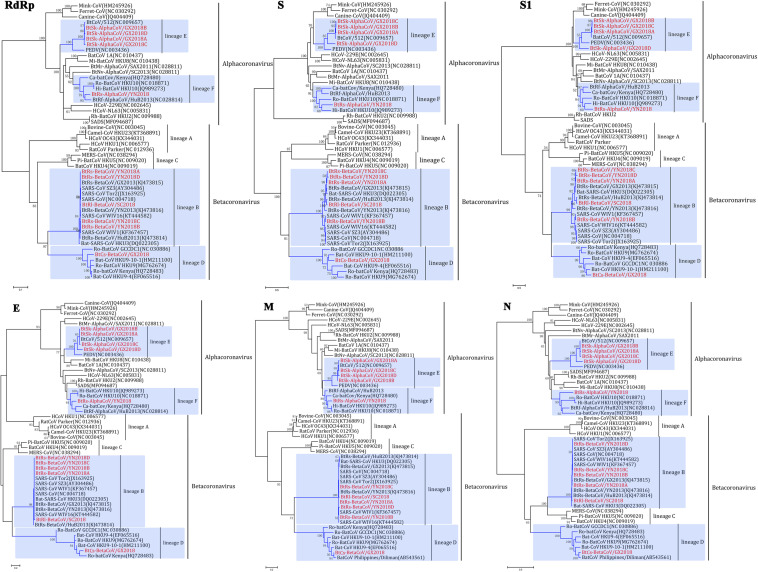
Phylogenetic trees based on aa sequences of RdRp, S, S1, E, M, and N. The trees were constructed by the maximum likelihood method using appropriate models (WAG + G + I for RdRp and N; LG + G + I for S, S1, and E; rtREV + G for M) with bootstrap values determined by 1000 replicates. Only bootstraps >40% are shown. The scale bars represent 0.05 (RdRp), 0.5 (S and S1), and 0.2 (E, M, and N), respectively.

To determine further the evolutionary relationships between these CoVs, phylogenetic trees were constructed using the aa sequences of RdRp, S, S1, E, M and N (Figure 4). As we have speculated before, α-GX2018A, α-GX2018B, α-GX2018C, α-GX2018D and α-YN2018 clustered with the genus *Alphacoronavirus*, and β-SC2018, β-YN2018A, β-YN2018B, β-YN2018C, β-YN2018D, and β-GX2018 clustered with the genus *Betacoronavirus*. The results of the phylogenetic analyses were consistent with those of the sequence identity analyses, and confirmed that the identified CoVs could be divided into four lineages.

#### Lineage E

α-GX2018A, α-GX2018B and α-GX2018C clustered with BtCoV/512, and their branches were short, reflecting the high sequence similarities. In addition, phylogenetic analysis of the S protein supported our previous analysis that α-GX2018D represented an independent clade between PEDV and BtCoV/512. Further analysis of S1 revealed that α-GX2018D represented a separate evolution distant from all other members of lineage E. From the length of the branch, α-GX2018D had a closer relationship with PEDV, which means that α-GX2018D may have undergone recombination with the ancestors of PEDV and BtCoV/512.

#### Lineage F

α-YN2018 clustered with Hi-batCov HKU10, Ro-BatCoV HKU10, Ca-batCoV/Kenya, and α-HuB2013. However, α-YN2018 had closer relationships with α-HuB2013 of *R. ferrumequinum* in RdRp and N, and closer relationships with HKU10 of *Hipposideros* and *Rousettus* bats in S and M. This means that recombination may have occurred between α-YN2018 and these three related CoVs.

#### Lineage B

β-SC2018, β-YN2018A, β-YN2018B, β-YN2018C, and β-YN2018D clustered with SARS- and SARSr- CoVs under lineage B. In RdRp, E, M, and N trees, these novel SARSr-CoVs and SARS-CoVs had short branches, which indicated that they were closely related in these regions. Phylogenetic analysis of S and S1 revealed that β-YN2018B was closer to SARS-CoV SZ3 and SARS-CoV Tor2 as well as WIV1 and WIV16.

#### Lineage D

β-GX2018 of *C. sphinx* clustered with HKU9-related CoVs of *Rousettus* and GCCDC1-related CoVs of *Eonycteris* under lineage D; however, β-GX2018 always represented a separate lineage distinct from other members of lineage D. Although BatCoV Philippines/Diliman of *C. brachyotis* had a closer relationship with α-YN2018 in M and N, it was only partially sequenced, so we could not analyze its evolutionary relationship with α-YN2018 in RdRp, S, and E, which hindered further analysis.

### Recombination Analysis

**FIGURE 5:**
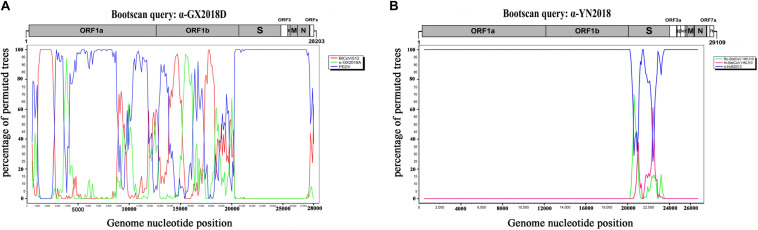
Detection of potential recombination events by bootscan analysis. **(A)** Full-length genome sequence of α-GX2018D was used as a query sequence and BtCoV/512, α-GX2018A, and PEDV as reference sequences. **(B)** Full-length genome sequence of α-YN2018 was used as a query sequence and Ro-BatCoV HKU10, Hi-batCov HKU10, and α-HuB2013 as reference sequences. All analyses were performed with a Kimura model, a window size of 1000 base pairs, and a step size of 100 base pairs. The gene map of query genome sequences is used to position breakpoints.

We found that recombinant events had occurred among α-GX2018D, other bat CoVs, and PEDV of lineage E. α-GX2018D showed the highest degree of similarity to BtCoV/512 in the ORF1ab region, and the highest degree of similarity to PEDV in the S1 region. Evolutionary analysis showed that the S of α-GX2018D may have acted as the common ancestors of BtCoV/512 and PEDV. We analyzed possible recombination events in lineage E using SimPlot software. Similarity plot and bootscan results demonstrated that the S-N region of the α-GX2018D had the highest degree of similarity to PEDV, and complicated recombination may have happened between bat CoV and PEDV in the ORF1ab region (Figure 5A).

For CoVs of lineage F, at least two recombination events had occurred (Figure 5B). Similarity plot and bootscan analysis showed that the S1 subunits of the α-YN2018 had the highest degree of similarity to Ro-BatCoV HKU10, while their S2 subunits had the highest degree of similarity to Hi-batCov HKU10 (Fig 5B). The complicated recombination history between these alpha-CoVs suggests frequent gene transfers, especially of S, among different CoVs, which may be the result of the cross-species transmission of these CoVs.

## Discussion

The identification of SARSr-CoV in bats in 2005 first established a genetic relationship between bats and human SARS-CoVs (Lau et al., 2005; Li et al., 2005). After that, in the following 13 years, the constant discovery of bat SARSr-CoVs suggested the risk of re-emergence of SARS-CoV originating from bat species. Other than that, two CoV-related cases, human MERS in 2012 and SADS in 2017, which had a great impact on health and the economy were identified as being of bat origin (Omrani et al., 2015; Zhou et al., 2018). In our previous study, a large-scale virome analysis was conducted between 2010 and 2013 to understand the ecological diversity of bat viruses and the bat origin of emerging infectious diseases. *Rhinolophus* bats in Yunnan and Guangxi Provinces have provided genetic clues to the origin of SARS-CoV, and a SADS-CoV- and HKU2-related CoV, BtRf-alphaCoV/YN2012, has also been found in Yunnan Province (Wu et al., 2016a). Sichuan Province has diverse wildlife resources and the largest pig breeding in China, and a MERS-related CoV was reported in bats in this province in 2013 (Yang et al., 2014). It is necessary for us to conduct continuous surveillance for the prevalence, genetic diversity and geographical distribution of CoVs in some bat species in these potential high-risk regions. Using virome analysis, our study investigated the presence and genetic diversity of CoVs circulating in bat species from Sichuan, Yunnan, and Guangxi Provinces between 2016 and 2017.

The close relationship between five alpha-CoVs in *S. kuhlii* in Guangxi Province and a previously reported CoV in *S. kuhlii* in Hainan Province reveals that this bat species from different geographic locations contained the same CoV species, but with distinct S proteins. The S1 region of α-GX2018D is more closely related to that of PEDV than that of any other bat CoV, and is phylogenetically located at the root of the lineage of PEDV and *S. kuhlii* CoVs. This finding suggests the presence of a much closer common ancestor of PEDV in bats CoVs. The recombination analysis supports that the S region of PEDV may come from the ancestor of α-GX2018D. Considering that bat species such as *S. kuhlii* prefers to circulate around farmhouses and pigsties, and other PEDV-related sequences have been reported in bats in previous reports (Simas et al., 2015; Phan et al., 2018), the relationship between bat CoVs and PEDV should be further investigated.

The discovery of β-SC2018 provides evidence that *Rhinolophus* in Sichuan Province could harbor SARSr-CoVs, and reveals a broader geographical distribution of these viruses. The discovery of β-YN2018B here, combined with previously reported WIV1 and WIV16 (Ge et al., 2013; Yang et al., 2015), revealed that these ACE2- adaptable SARSr-CoVs were continuously circulating in *R. sinicus* of Yunnan Province at least from 2012 to 2016. These findings emphasize again the importance of continuous longitudinal monitoring for SARSr-CoVs in *Rhinolophus* over a wide area. Although no SADS and HKU2-related CoV were found in *Rhinolophus* bats in our study, the surveillance of this CoV type in bats is still needed in the future.

In our previous study, by the identification of correlated alpha-CoVs in different Miniopterus species from multiple regions, the characteristics of co-infection, recombination, and host-shifting for alpha-CoVs have been described. Here, the alpha-CoVs of lineage F found in more diverse bat species (including frugivorous and insectivorous bats) extends the known host range of these viruses. However, the recombination events found between α-YN2018, Ro-BatCoV HKU10, and Hi-batCov HKU10, and the multiple origins of the S of α-YN2018, further indicate that a more distant host-shifting in accordance with the recombination of the S region may have happened in the evolutionary history of these viruses among diverse host species.

When we collected *S. kuhlii* in Guangxi Province, we found that there were many *C. sphinx* at the sampling site. The *Pteropodidae* bats (*C. sphinx* bat belongs to *Pteropodida*e) are natural hosts of several emergent human pathogens such as Hendra virus, Nipah virus, and Ebola virus (Leroy et al., 2005; Halpin et al., 2011; Sendow et al., 2013; Mari Saez et al., 2015; Paez et al., 2017). An Ebola-related filovirus was found in the Chinese *Pteropodidae* bats recently (Yang et al., 2019), and thus, we collected samples of *C. sphinx* for virome analysis. The *C. sphinx* beta-CoV, β-GX2018, represents an evolved independent lineage different from HKU9 of *Rousettus* and GCCDC1 of *Eonycteris* under lineage D. Based on the partially sequenced region, CoV strain Philippines/Diliman1525G2/2008 previously identified in *C. brachyotis* from the Philippines, could also be clustered into this separate β-GX2018 clade. By providing full-length sequence data, this finding reveals a new host range of this virus in an unreported location.

## Author Contributions

YH, QJ, and ZW conceived the experiments, analyzed the results, and wrote the manuscript. YH, JD, HS, and ZW conducted the experiments and analyzed the results. JZ, GZ, and SZ collected the specimens. All authors reviewed the manuscript.

## Conflict of Interest Statement

The authors declare that the research was conducted in the absence of any commercial or financial relationships that could be construed as a potential conflict of interest.
