# Corrigendum: Identification of Diverse Bat Alphacoronaviruses and Betacoronaviruses in China Provides New Insights Into the Evolution and Origin of Coronavirus-Related Diseases

**DOI:** 10.3389/fmicb.2020.597510

**Published:** 2020-10-26

**Authors:** Yelin Han, Jiang Du, Haoxiang Su, Junpeng Zhang, Guangjian Zhu, Shuyi Zhang, Zhiqiang Wu, Qi Jin

**Affiliations:** ^1^NHC Key Laboratory of Systems Biology of Pathogens, Institute of Pathogen Biology, Chinese Academy of Medical Sciences and Peking Union Medical College, Beijing, China; ^2^Key Laboratory of Zoonosis of Liaoning Province, College of Animal Science and Veterinary Medicine, Shenyang Agricultural University, Shenyang, China; ^3^EcoHealth Alliance, New York, NY, United States

**Keywords:** bats, coronaviruses, porcine epidemic diarrhea virus, severe acute respiratory syndrome coronavirus, ecological and genetic diversity

In the original article, there was a mistake in Table 1 as published. All instances of “*Rhinolophus affinis*” in Table 1 should be corrected into “*Rhinolophus sinicus*.” The corrected Table 1 appears below.

**Table 1:** Coronavirus distribution in different bat species and locations.

**Province**	**Species**	**Pool code for NGS**	**Collection date [year.month]**	**Virus detection rate (pos. /indiv. [%])**	**Sequencing reads related to CoV**	**Virus**
Sichuan	*Rhinolophus* spp.	55	2016.08	2/22 [9.1%]	1598	BtRl-BetaCoV/SC2018
Yunnan	*Rhinolophus sinicus*	57	2016.09	2/39 [5.1%]	84818	BtRs-BetaCoV/YN2018A
	*Rhinolophus sinicus*	60	2016.09	4/40 [10%]	884839	BtRs-BetaCoV/YN2018BBtRs-AlphaCoV/YN2018
	*Rhinolophus sinicus*	61	2016.09	2/46 [4.3%]	37997	BtRs-BetaCoV/YN2018C
	*Rhinolophus sinicus*	62	2016.09	4/51 [7.8%]	11131	BtRs-BetaCoV/YN2018D
Guangxi	*Scotophilus kuhlii*	3	2017.05	2/27 [7.4%]	5609	BtSk-AlphaCoV/ GX2018A
	*Scotophilus kuhlii*	4	2017.05	1/39 [2.6%]	4381	BtSk-AlphaCoV/GX2018D
	*Cynopterus sphinx*	11	2017.05	2/30 [6.7%]	6138	BtCs-BetaCoV/GX2018
	*Scotophilus kuhlii*	12	2017.05	1/41 [2.4%]	4353	BtSk-AlphaCoV/GX2018B
	*Scotophilus kuhlii*	13	2017.05	2/43 [4.7%]	37	BtSk-AlphaCoV/GX2018C

In the original article, there was an error. All instances of “*Rhinolophus affinis*” or “*R. affinis*” in the text should be corrected into “*Rhinolophus sinicus*” or “*R. sinicus*.”

The authors apologize for this error and state that this does not change the scientific conclusions of the article in any way. The original article has been updated.

