# Supplementary material for: Identification of Diverse Bat Alphacoronaviruses and Betacoronaviruses in China Provides New Insights Into the Evolution and Origin of Coronavirus-Related Diseases
Source: Front Microbiol. 2019 Aug 14;10:1900. doi: 10.3389/fmicb.2019.01900 (PMC6702311; doi:10.3389/fmicb.2019.01900)
Supplement: Supplementary file 1 [file Data_Sheet_1.PDF]

Supplementary Table 1A. Transcription regulatory sequences (TRSs) for α-GX2018A, α-GX2018B and α-GX2018C.

Coronavirus	ORF	TRS location (CoV) (nt)	Leader TRS region and intergenic TRS (CoV)	Distance from TRS to AUG (nt)
α-GX2018A	ORF1ab	71-76	UUCA **CUAAA** GAAAU	219
	S	20,669-20,674	UUCA **CCAAA G**	-2
	ORF3	24,753-24,758	UUCA **CAUUA** GAACC	29
	E	25,433-25,438	UUCA **CUAGA** GAAU**AU**	4
	M	25,670-25,675	CAAG **AUAAA** GAAA**AU**	4
	N	26,363-26,368	UUAG **CUAAA** AGAAA	10
	ORFx	27,568-27,573	UUCA **CUAAA** CA**AUG**	2
α-GX2018B	ORF1ab	35-40	UUCA **CUAAA** GAAAU	219
	S	20,633-20,638	UUCA **CCAAA G**	-2
	ORF3	24,720-24,725	UUCA **CAUUA** GAACC	29
	E	25,400-25,405	UUCA **CUAGA** GAAU**AU**	4
	M	25,637-25,642	CAAG **AUAAA** GAAA**AU**	4
	N	26,330-26,335	UUAG **CUAAA** AGAAA	8
	ORFx	27,530-27,535	UUCA **CUAAA** CA**AUG**	2
α-GX2018C	ORF1ab	32-37	UUCA **CUAAA** GAAAU	218
	S	20,629-20,634	UUCA **CCAAA G**	-2
	ORF3	24,716-24,721	UUCA **CAUUA** GAACC	29
	E	25,396-25,401	UUCA **CUAGA** GAAU**AU**	4
	M	25,633-25,638	CAAG **AUAAA** GAAA**AU**	4
	N	26,326-26,331	UUAG **CUAAA** AGAAA	8
	ORFx	27,531-27,536	UUCA **CUAAA** CA**AUG**	2

Supplementary Table 1B. Transcription regulatory sequences (TRSs) for β-SC2018, β-YN2018A, β-YN2018C and β-YN2018D.

Coronavirus	ORF	TRS location (CoV) (nt)	Leader TRS region and intergenic TRS (CoV)	Distance from TRS to AUG (nt)
β-SC2018[e]	ORF1ab	64-69	UCUA **ACGAA** UUUAA	191
	S	21,449-21,454	ACUA **ACGAA** **AUG**	0
	ORF3a	25,181-25,186	CAUA **ACGAA** UU**AUG**	3
	E	26,031-26,036	UGAG **ACGAA** UU**AUG**	2
	M	26,270-26,275	UCUA **ACGAA** UAACUA	44
	ORF6	26,835-26,840	ACAU **ACGAA** GCUUU	155
	ORF7a	27,184-27,189	UCUA **ACGAA** **AUG**	0
	ORF8a	27,690-27,695	UCUA **ACGAA** **AUG**	0
	N	28,062-28,067	UCUA **ACGAA** AAACU	8
β-YN2018A[f]	ORF1ab	68-73	UCUA **ACGAA** UUUAA	192
	S	21,488-21,493	ACUA **ACGAA** **AUG**	0
	ORF3a	25,223-25,228	CAUA **ACGAA** UU**AUG**	3
	E	26,073-26,078	UGAG **ACGAA** UU**AUG**	2
	M	26,312-26,317	UCUA **ACGAA** UAACUA	44
	ORF6	26,877-26,882	ACAU **ACGAA** GCUUU	155
	ORF7a	27,231-27,236	UCUA **ACGAA** **AUG**	0
	ORF8a	27,737-27,742	UCUA **ACGAA** **AUG**	0
	N	28,109-28,114	UCUA **ACGAA** AAACU	8
β-YN2018C[h]	ORF1ab	65-70	UCUA **ACGAA** UUUAA	192
	S	21,484-21,489	ACUA **ACGAA** **AUG**	0
	ORF3a	25,216-25,221	CAUA **ACGAA** UU**AUG**	3
	E	26,066-26,071	UGAG **ACGAA** UU**AUG**	2
	M	26,305-26,310	UCUA **ACGAA** UAACUA	44
	ORF6	26,870-26,875	ACAU **ACGAA** GCUUU	155
	ORF7a	27,224-27,229	UCUA **ACGAA** **AUG**	0
	ORF8	27,730-27,735	UCUA **ACGAA** **AUG**	0
	N	28,102-28,107	UCUA **ACGAA** AAACU	8
β-YN2018D[i]	ORF1ab	67-72	UCUA **ACGAA** UUUAA	192
	S	21,486-21,491	ACUA **ACGAA** **AUG**	0
	ORF3a	25,218-25,223	CAUA **ACGAA** UU**AUG**	3
	E	26,068-26,073	UGAG **ACGAA** UU**AUG**	2
	M	26,307-26,312	UCUA **ACGAA** UAACUA	44
	ORF6	26,872-26,877	ACAU **ACGAA** GCUUU	154
	ORFx	27,227-27,232	GAUA **ACGAA** CACU**AUG**	4
	ORF7a	27,747-27,752	UCUA **ACGAA** **AUG**	0
	ORF8	28,253-28,258	UCUA **ACGAA** **AUG**	0
	N	28,625-28,630	UCUA **ACGAA** AAACU	8

Supplementary Table 2. Comparison of genomic features and amino acid identities between the four alpha-CoVs and representative members of lineage E.

Virus	Nucleotide or amino acid	α-GX2018A	α-GX2018B	α-GX2018C	α-GX2018D
BtCoV/512	Genome	97	97	97	97
	ORF1a	96.6	96.6	97.1	96
	ORF1a (1000-2500nt)	81.3	82	93.7	94.6
	ORF1b	99.6	99.6	99.2	99.4
	RdRp	99.4	99.7	99.7	99.7
	S	93.4	96.1	96.2	61
	S1	90.2	95.3	95.3	39.7
	ORF3	99.1	99.1	96.9	93.4
	E	100	100	98.7	84.8
	M	99.6	97.3	98.2	92.2
	N	98.2	98.7	98.0	79.3
	ORFx	65.7	67.6	67.6	69
PEDV	Genome	77	77	77	77
	ORF1a	62.6	62.5	62.5	62.3
	ORF1b	86.8	86.7	86.6	86.4
	RdRp	51.1	51.4	51.4	51.4
	S	60.4	60.4	60.5	60
	S1	46.7	46.9	46.9	42.7
	ORF3	58.9	58.9	58.9	57.3
	E	77.6	77.6	78.9	73.4
	M	80.4	81.8	80.9	77.7
	N	52.2	52.6	52.2	64.9
	ORFx	28.1	25	25	30.6

Supplementary Table 3. Comparison of genomic features and amino acid identities between the α-YN2018 and representative members of lineage F.

Virus	Nucleotide or amino acid	Hi-BatCoV HKU10	Ro-BatCoV HKU10	AlphaCoV/ HuB2013	Ca-batCov/Kenya
α-YN2018	Genome	77%	78%	79%	—
	ORF1a	70.3	70.7	72.7	—
	ORF1b	89.1	89.1	92.4	89.1
	RdRp	87.1	87.1	89.8	86
	S	57.3	56	54.3	55.6
	ORF3a	55.7	49.3	51.4	60.6
	ORF3b	—	—	—	—
	ORF3c	—	—	—	—
	E	74.3	74.3	73.8	67.6
	M	85.7	84.9	84.6	81.9
	N	54.9	55	63.9	57.6
	ORF7a	13.3	22	25	16
	ORF7b	50	20	—	—

Supplementary Table 4. Comparison of genomic features and amino acid identities between the five SARSr-CoVs and representative members of lineage B.

Virus	Nucleotide or amino acid	β-SC 2018	β-YN 2018A	β-YN 2018B	β-YN 2018C	β-YN 2018D
SARS SZ3	Genome	92	96	96	97	97
	ORF1a	94.2	98	98.1	98.1	98.2
	ORF1b	98.5	99.5	99.4	99.4	99.4
	RdRp	99.4	99.7	99.6	99.5	99.7
	S	78.9	78.1	92.5	78	78.2
	ORF3a	88.3	89.4	96	89.1	95.3
	ORF3b	93.9	93.9	94.7	94.7	94.7
	E	98.7	100	100	100	100
	M	98.6	98.2	97.7	99.5	97.3
	ORF6	100	98.4	93.7	98.4	93.7
	ORF7a	91.8	95.9	94.3	95.1	94.3
	ORF7b	93.2	93.2	93.2	93.2	93.2
	ORF8	32.6	32.6	34.1	34.1	33.3
	N	97.6	98.8	99.8	99.8	99.8
SARS WIV1	Genome	93	98	98	98	92
	ORF1a	94.4	97	99	99	99.1
	ORF1b	98.6	99.7	99.8	99.8	99.6
	RdRp	99.5	99.8	99.9	99.8	99.8
	S	79.1	78.9	98.8	78.8	79
	ORF3a	89.4	90.5	98.2	90.1	97.1
	ORF3b	94.7	94.7	95.6	95.6	95.6
	E	98.7	100	100	100	100
	M	97.3	99.5	100	98.2	100
	ORF6	100	95.2	100	95.2	100
	ORFx	—	—	98.8	—	98.8
	ORF7a	96.7	97.5	99.2	98.4	99.2
	ORF7b	97.7	100	100	100	100
	ORF8	91.7	95.9	100	100	99.2
	N	97.4	98.6	99.5	99.5	99.5
SARS WIV16	Genome	93	93	99	93	95
	ORF1a	94.5	99	99.1	99.1	99.2
	ORF1b	98.5	99.3	99.3	99.2	99.3
	S	79.4	78.8	93.4	78.7	78.8
	ORF3a	89.8	90.9	98.5	90.5	97
	ORF3b	94.7	94.7	95.6	95.6	95.6
	E	98.7	100	100	100	100
	M	97.3	99.5	100	98.2	100
	ORF6	96.7	93.7	98.4	93.7	98.4
	ORFx	—	—	98.8	—	98.8
	ORF7a	96.7	97.5	99.2	98.4	99.2
	ORF7b	97.7	100	100	100	100
	ORF8	90.9	95	99.2	99.2	100
	N	97.4	98.3	99.3	99.3	99.8

Supplementary Table 5. Comparison of genomic features and amino acid identities between the β-GX2018 and representative members of lineage D.

Virus	Nucleotide or amino acid	Ro-BatCoV HKU9	Ro-BatCoV GCCDC1	BatCoV HKU9-4	BatCoV HKU9-10-1	BatCoV Philippines/Diliman
β-GX2018	Genome	80	72	79	78	—
	ORF1a	75.8	67.1	76.8	75.7	—
	ORF1b	90.7	84.9	90.8	90.6	—
	RdRp	86.7	88.6	88.7	85.2	—
	S	62.9	64.9	64.3	73	—
	ORF3	62.7	51	57	61.8	—
	E	85.3	64.5	88	88.2	—
	M	87.1	79	89.6	89.8	96
	N	79.8	64.3	79.6	81.3	91
	ORF7a	24.7	24.3	39.7	37.1	80
	ORF7b	29.4	16.7	29.0	37.5	84.3
	ORF7c	—	25.2	—	—	80.8
